# Randomized clinical trials and informed consent in pediatric oncology: a Nordic comparative study of parents’ experiences

**DOI:** 10.3389/fped.2026.1819033

**Published:** 2026-05-14

**Authors:** Anna Schröder Håkansson, Ann-Christine Andersson, Karin Mellgren, Nina Mogensen, Jonas Abrahamsson, Margaretha Stenmarker

**Affiliations:** 1 Department of Pediatrics, Institute of Clinical Sciences, Sahlgrenska Academy, University of Gothenburg, Gothenburg, Sweden; 2 Department of Pediatric Oncology, Queen Silvia Children’s Hospital, Sahlgrenska University Hospital, Gothenburg, Sweden; 3 Jönköping Academy for Improvement of Health and Welfare, School of Health and Welfare, Jönköping University, Jönköping, Sweden; 4 Region Jönköping County, Jönköping, Sweden; 5 Department of Pediatric Oncology, Astrid Lindgren Children’s Hospital, Karolinska University Hospital, Stockholm, Sweden; 6 Department of Women’s and Children’s Health, Childhood Cancer Research Unit, Karolinska Institutet, Stockholm, Sweden; 7 Department of Pediatrics and Futurum, Region Jonkoping County, Jönköping, Sweden; 8 Department of Biomedical and Clinical Sciences, Linköping University, Linköping, Sweden

**Keywords:** acute myeloid leukemia, informed consent, multimethod approach, non-Hodgkin lymphoma, parents experience, pediatric oncology, randomized clinical trials, thematic analyses

## Abstract

**Background:**

In pediatric oncology, randomized clinical trials are an integral part of standard treatment. Because enrollment typically must occur before therapy begins, families receive trial information shortly after diagnosis and must provide consent within a brief time frame. The informed consent process therefore often takes place while parents are experiencing emotional stress, have limited medical knowledge, and have little opportunity to reflect on or discuss the available treatment options, as stipulated by good clinical practice.

**Objective:**

To gain insight into parents’ experiences of the informed consent process and their motivations for participating in a randomized clinical trial.

**Methods:**

A Nordic survey using a study-specific questionnaire was conducted. Parents of children enrolled in the NOPHO-DBH AML 2012 and B-NHL 2013 protocols responded. A multimethod approach was applied. Quantitative data were analyzed using descriptive statistics and factor analysis, while qualitative data were analyzed thematically.

**Results:**

In total, parents of 72 children (60 single mothers, 32 single fathers, and 8 couples) participated, yielding 100 complete questionnaires. The two protocol groups were similar in sample size, with 49 participants from AML 2012 and 51 from B NHL 2013. Factor analysis identified three factors and one single-item indicator. No statistically significant differences were found between the protocols for any of the variables examined: Influence of information provided (Factor 1; *p* = .570), Emotional influences (Factor 2; *p* = .308), Influence on decision-making (Factor 3; *p* = .017), and Perceived impact on care in the event of non-participation (Indicator; *p* = .174). The qualitative results illuminate the parents’ motivation for enrollment which comprise three themes: Opportunities and risks associated with new treatment, Contributing to research and helping others, Information and strain in the situation.

**Conclusion:**

This study contributes to a broader understanding of parents’ experiences of the informed consent process within the context of standard treatment practice in pediatric oncology. Despite making decisions under considerable emotional pressure, most parents reported satisfaction with the information provided and did not express regret regarding their enrollment decision. The findings underscore the importance of communication practices that support parents’ comprehension and decision-making during the consent process.

## 1 Introduction

Clinical trials (CT) are one of the cornerstones behind the increasing survival rate in pediatric oncology (1–3). Randomized clinical trials (RCT), in which participants are randomly assigned to different treatment groups, are widely regarded as the gold standard for evaluating new treatments. The conduct of all clinical trials is guided by the principles of Good Clinical Practice (GCP), which serve as the overarching framework for ensuring ethical standards, participant protection, and the reliability of study data (4). A majority of children with cancer in countries with higher resource settings are treated within a clinical trial (5–7). This is an important part in testing new ways to treat pediatric cancers, aiming to improve survival and quality of life, by finding safer, more effective therapies (1).

Most pediatric cancers need prompt treatment, and the recommendation is to begin treatment as soon as diagnosis is established (8). Consequently, information about the diagnosis and treatment plan must be communicated promptly. If the child is to be enrolled in an RCT, the family is required to give informed consent within a short timeframe, often with limited opportunity to reflect on or discuss the available options before treatment begins (4). The informed consent process (ICP) is challenging and stressful for all involved parties, particularly for the child and the parents but also for the pediatric oncologist (PO), who must ensure that information is received and understood by the family. In most cases, the family has limited experience of cancer diagnosis and treatment including RCT (9). Parents often struggle to understand the information due to emotional stress and limited medical knowledge and as a result they may find it difficult to take appropriate actions (10–12). In addition, an inherent challenge arises from the need to maintain parental comprehension of the information materials while the sponsor, in order to mitigate regulatory and legal exposure, requires an exhaustive delineation of all reasonably foreseeable adverse events that might confer liability (13). However, allowing their child to enroll in a RCT can give parents a sense of actively doing something for their child (14). Depending on the severity of symptoms and the child's age, the child is often involved in the ICP (15); However, the decision is typically made by the parent, a pattern that also holds in studies from countries where older children are invited to participate in the decision-making process, as these children frequently delegate the final decision to their parent (16).

In the Nordic countries, both acute myeloid leukemia (AML) and Mature B-cells lymphoma (B-NHL), are among the diseases that have been treated by phase III RCTs, AML according to NOPHO-DBH AML 2012: A phase III randomized trial in children with acute myeloid leukemia (AML 2012) and B-NHL according to B-NHL 2013: A phase III randomized trial in children and adolescents with B-cell non-Hodgkin lymphoma (B-NHL 2013). While these protocols were open, the nationally recommended first line treatments of AML and B-NHL constituted the standard arms of these RCTs. This approach could make it difficult for parents to clearly distinguish between participation in the RCT and the recommended therapy (17). Even if most children with cancer in the Nordic countries are treated according to common guidelines, which often are designed as a CT and in many cases as an RCT (18), there are limited descriptions from the Nordic countries on the parents' experiences of enrolment and participation in a phase III RCT (15, 19).

The present study was undertaken to investigate the ICP, for participation in phase III RCTs, in pediatric oncology in the Nordic countries. The study focused on the perspective of parents whose children were enrolled in AML 2012 or B-NHL 2013. The aim was to gain insight into parents' experiences of the ICP and their motivation for participating.

## 2 Materials and methods

### 2.1 Design

This Nordic study had a cross-sectional design with a multimethod approach (20), and investigated parental experiences through a survey including both quantitative and qualitative data. This study is a sub-study conducted within the framework of two prospective studies on health-related quality of life (HRQoL): one involving children with AML (HRQoL AML) and one involving children with B-NHL (HRQoL B-NHL).

### 2.2 Setting and sample

Parents of children diagnosed with AML treated according to NOPHO-DBH AML 2012: A phase III randomized trial in children with acute myeloid leukemia. (EudraCT2012-002934-35 and clinicaltrials.gov ID NCT01828489), from the year 2013 to 2024 and parents of children diagnosed with B-NHL treated according to B-NHL 2013*:* A phase III randomized trial in children and adolescents with B-cell non-Hodgkin lymphoma. (EudraCT 2013-003253-21 and clinicaltrials.gov ID NCT03206671) from the year 2017 to 2025, were invited. These two RCTs share the same study design, randomizing treatment elements upfront in the protocol and with short total treatment periods, i.e., up to 6 months. Each parent received information regarding their child's diagnosis and specific treatment plan, including the relevant RCT, from a physician at a pediatric oncology department in their respective country. The informed consent was signed by the parents before any treatment specific for the RCT was started. In line with GCP guidelines, both protocols AML 2012 and B-NHL 2013 contained age-adapted information materials for the children. Participating countries were Sweden, Norway, Denmark and Finland (Tables 1–3).

**Table 1:** Characteristics of the parents (mothers and fathers).

Age years Mothers (*n* = *n* 72)						
**Protocol**	AML 2012		B-NHL 213		Total	
Age years Mothers (*n* = 72)						
Mean	37,85		40,21		39,10	
Median	37,5		41		38,50	
Range	28–49		27–57		27–57	
Age years Fathers (*n* = 68 missing 4)						
Mean	40,68		43,82		42,25	
Median	41		44,50		41,50	
Range	28–51		28–55		28–55	
Country of Birth in numbers						
	AML 2012		B-NHL 2013		Total	
	Mothers	Fathers	Mothers	Fathers	Total	
Sweden	12	12	12	11	47	
Norway	7	3	0	0	10	
Finland	13	16	21	21	71	
Denmark	0/0	0	2	3	5	
Other	2	3	3	3	11	
Education	*n* (%)					
	AML 2012		B-NHL 2013		Total	
	Mothers	Fathers	Mothers	Fathers	Mothers	Fathers
Compulsory education	3 (8,8)	3 (8,8)	6 (15,8)	7 (18,4)	9 (12,5)	10 (13,9)
Upper secondary education	9 (26,5)	9 (26,5)	10 (26,3)	11 (28,9)	19 (26,4)	20 (27,8)
Post-secondary education	22 (64,7)	22 (64,7)	7 (18,4)	15 (39,5)	44 (61,1)	37 (55,4)
Unknown	0	0	0	5 (13,2)	0	5 (6,9)

**Table 2:** Characteristics of participants (mothers, fathers, couples).

**Participants**		AML 2012		B-NHL 2013		Total	
		*n*	%	*n*	%	*n*	%
Mothers		32	(65,3)	28	(54,9)	60	(60)
Fathers		15	(30,6)	17	(33,3)	32	(32)
Couples		2	(4,1)	6	(11,8)	8	(8)
Total		*n* = 49		*n* = 51		*n* = 100	
Time of responds		AML2012		B-NHL 2013		Total	
During treatment		7	(14,3)	16	(31,4)	23	(23)
EOT up 2 months after EOT		11	(46,9)	23	(45,1)	34	(34)
2 months to 12 months after EOT		23	(34,7)	12	(23,5)	35	(30,2)
1–2 years after EOT		0	0	0	0	0	0
More than 2 years after		8	(16,3)	0	0	8	(8)
Participants in relation to the child's age							
		AML2012		B-NHL 2013		Total	
Mothers		14	(82,4)	3	(17,6)	16	(26,7)
Fathers	2–4 years	7	(77,8)	2	(22,2)	3	(42,9)
Couples		1	(33,3)	2	(66,7)	1	(20)
Mothers		5	(50)	5	(50)	10	(16,7)
Fathers	5–7 years	2	(25)	6	(75)	2	(28,6)
Couples		1	(25)	3	(75)	3	(60)
Mothers		7	(38,9)	11	(61,1)	20	(33,3)
Fathers	8–12 years	4	(40)	6	(60)	2	(28,6)
Couples		0	0	0	0	0	(28,6)
Mothers	13–18 years	6	(40)	9	(60)	14	(23,3)
Fathers		1	(25)	3	(75)	2	(28,6)
Couples		0	0	1	(100)	1	(20)

EOT, end of treatment.

**Table 3:** Children characteristics.

	AML 2012		B-NHL 2013		Total	
	*n*	%	*n*	%	*n*	%
Total	34	(47,2)	38	(52,8)	72	(100)
Gender						
Male	16	(47,1)	30	(78,9)	46	(63,9)
Female	18	(52,9)	8	(21,1)	26	(36,1)
Age by group						
2–4 years	13	(38,2)	4	(10,5)	17	(23,6)
5–7 years	6	(17,6)	10	(26,3)	16	(22,2)
8–12 years	9	(26,5)	13	(34,2)	22	(30,6)
13–18 years	6	(17,6)	11	(28,9)	17	(23,6)
Country						
Sweden	14	(41,2)	13	(34,2)	27	(37,5)
Norway	8	(23,5)	0	0	8	(11,1)
Finland	12	(35,3)	21	(55,3)	33	(45,8)
Denmark	0	0	4	(10,5)	4	(5,6)
Randomizing						
No	5*	(14,7)	16*	(42,1)	21*	(29,2)
Yes	29	(85,3)	22	(57,9)	51	(70,8)

* Not eligible for the randomizing part in the protocol.

### 2.3 Study-specific questionnaire

The study-specific questionnaire was developed by Mogensen (19). The questionnaire consisted of demographic data, nine items which could be responded with YES, NO or NOT SURE and two open-ended questions (Tables 4–7). The nine items were related to the parental experiences of the ICP before enrolment in the RCT. The questionnaire was translated into all participating countries' languages by authorized translators.

**Table 4:** Influence of given information (factor 1).

Question/Item							
1. Did you get all the information you need to make a good decision about participating in the randomized study?							
Protocol	AML 2012	B-NHL 2013	Total	F 1	F 2	F 3	F4
	*n* (%)	*n* (%)	*n* (%)				
Yes	43 (87,5)	43 (84,3)	86 (86)	,739	,089	,327	−,121
No	3 (6,1)	3 (5,9)	6 (6)				
Not sure	3 (6,1)	5 (9,8)	8 (8)				
No answer/Not applicable	0	0	0				
Total	49	51	100				
2. Did you understand the information that was given to you regarding the randomized study?							
Protocol	AML 2012	B-NHL 2013	Total	F 1	F 2	F 3	F4
	*n* (%)	*n* (%)	*n* (%)				
Yes	35 (71,4)	36 (70,6)	71 (71)	,867	,072	,035	,129
No	3 (6,1,2)	6 (11,8)	9 (9)				
Not sure	9 (18,4)	9 (17,6)	18 (18)				
No answer/Not applicable	2 (4,1)	0	2 (2)				
Total	49	51	100				
6. Were you satisfied with the entire information process concerning participation in a randomized study?							
Protocol	AML 2012	B-NHL 2013	Total	F 1	F 2	F 3	F4
	*n* (%)	*n* (%)	*n* (%)				
Yes	37 (75,5)	37 (72,5)	74 (74)	,837	,088	,189	−,129
No	3 (6,1)	6 (11,8)	9 (9)				
Not sure	7 (14,3)	7 (13,7)	14 (14)				
No answer/Not applicable	2 (4,1)	1 (2,0)	3 (3)				
Total	49	51	100				

**Table 5:** Emotional influences (factor 2).

Question/Item							
3. Did you feel any pressure to participate in the study?							
Protocol	AML 2012	B-NHL 2013	Total	F 1	F2	F3	F4
	*n* (%)	*n* (%)	*n* (%)				
Yes	1 (2)	5 (9,8)	6 (6)	−,076	,734	,016	−,208
No	47 (95,9)	42 (82.4)	89 (89)				
Not sure	1 (2)	3 (5,9)	4 (4)				
No answer/Not applicable	0	1 (2)	1 (1)				
Total	49	51	100				
7. Was your child included in the entire information process concerning participation in a randomized study?							
Protocol	AML 2012	B-NHL 2013	Total	F 1	F 2	F 3	F4
	*n* (%)	*n* (%)	*n* (%)				
Yes	20 (40,8)	16 (31,4)	36 (36)	,155	,738	,293	,210
No	25 (51)	30 (58,8)	55 (55)				
Not sure	4 (8,2)	5 (9,8)	9 (9)				
No answer/Not applicable	0	0	0				
Total	49	51	100				
8. Are you satisfied with the information given to your child?							
Protocol	AML 2012	B-NHL 2013	Total	F 1	F 2	F 3	F4
	*n* (%)	*n* (%)	*n* (%)				
Yes	26 (53,1)	32 (62,7)	58 (58)	,363	,580	−,326	−,002
No	2 (4,1)	6 (11,8)	8 (8)				
Not sure	13 (26,5)	9 (17,6)	22 (22)				
No answer/Not applicable	0/8 (16,3)	2 (3,9)/2 (3,9)	2 (2)/10 (10)				
Total	49	51	100				

**Table 6:** Influence on decision-making (factor 3).

Question/Item							
4. Did you have enough time to consider participation in the randomization study?							
Protocol	AML 2012	B-NHL 2013	Total	F1	F2	F3	F4
	*n* (%)	*n* (%)	*n* (%)				
Yes	44 (89,8)	33 (64,7)	77 (77)	,105	,089	,816	−,074
No	2 (4,1)	9 (17,6)	11 (11)				
Not sure	3 (6,1)	19 (17,6)	12 (12)				
No answer/Not applicable	0	0	0				
Total	49	51	100				
9. If you were asked to participate in a randomized study now - would you make the same decision again?							
Protocol	AML 2012	B-NHL 2013	Total	F 1	F 2	F 3	F4
	*n* (%)	*n* (%)	*n* (%)				
Yes	46 (93,9)	40 (78,4)	86 (86)	,384	−,024	,660	,126
No	1 (2,0)	2 (3,9)	3 (3)				
Not sure	2 (4,1)	9 (17,6)	11 (11)				
No answer/Not applicable	0	0	0				
Total	49	51	100				

**Table 7:** Perceived impact on care in the event of non-participation (indicator).

Question/Item								
5. Suppose that you have decided **not** to participate in the study - do you think that would have made any difference to your child's regular medical care?								
Protocol		AML 2012	B-NHL 2013	Total	I1	I2	I3	I4
		*n* (%)	*n* (%)	*n* (%)				
Yes		3 (6,1)	8 (15,7)	11 (11)	,066	−,045	,004	,956
No		34 (69,4)	27 (52,9)	61 (61)				
Not sure		12 (24,5)	16 (31,4)	28 (28)				
No answer/Not applicable		0	0	0				
Total		49	51	100				

### 2.4 Procedure

The HRQoL survey including questionnaire administration occurred in AML 2012 from the year 2012 to 2024 and in B-NHL from the year 2017 to 2025. In both studies, parents were asked to decide on participation in RCTs before the medical treatment was initiated. In the AML 2012 study, the parents were approached after 2 months of treatment with oral and written information about the present study regarding their perspectives of the ICP. In the B-NHL 2013 study, this information was included in both the written and oral information which were provided prior to the start of treatment. All parents gave written consent. The parents were given the option to complete the questionnaire either individually or jointly as couples, depending on their preferences. They received and responded to the questionnaire at the end of treatment, approximately six months after diagnosis. Ethical approvals, according to each participating countries' legal system, have been obtained.; Sweden: Dnr: 721-13, Dnr: 1186-18/2019-00814, and Dnr: 2020-02386. Norway: 2016/1710, Finland: HUS/2894/2019, EudraCT 2012-002934-35, EudraCT 2013-003253-21 and EudraCT 2023-5055509-18-00 Denmark: According to Danish legislation, the study did not need Research ethics committee approval.

### 2.5 Data analysis

Quantitative data were analyzed using descriptive statistics. Frequency and percentages were calculated for all items, and for demographic data. Statistical comparisons between protocols were conducted using Fisher's exact test to obtain valid p-values under sparse data conditions. An exploratory factor analysis was conducted to examine underlying structures between the different items in the questionnaire. The analysis was based on Principal Component Analysis with Varimax rotation and Kaiser normalization. For each factor, a composite variable was generated from the items that loaded onto that factor. The significance level was set at *p* < .05. The data were analyzed using IBM SPSS Version 30.0™.

Qualitative data were analyzed using brief text analysis inspired by Robinson (21). The two open-ended questions' transcripts were coded by the first author (ASH) using NVivo™. The authors (ASH, ACA, MS) independently coded the transcripts, reviewed discrepancies, and agreed upon consistent coding. The codes were abstracted into three themes and illustrated by quotations, which were translated to English by a professional language examiner. All three authors (ASH, ACA, MS) have experience in qualitative data analysis. ACA is a senior researcher with extensive expertise in qualitative methodological approaches and led the analytical work. Ultimately, all quantitative and qualitative findings were intertwined, inspired by a multimethod approach (20).

## 3 Results

### 3.1 Demographics

Parents of 72 children who were enrolled in AML 2012 and B-NHL 2013, responded to the questionnaire. The parents had the option to fill in the questionnaire together as a couple or individually. Altogether, 100 questionnaires were completed, comprising responses from 60 mothers, 32 fathers, and 8 couples. Of these were 49 from AML 2012 and 51 from B-NHL (Table 2).

The results present the responders both as a single group and as subgroups, AML 2012 and B-NHL 2013, categorized by the RCT. When only one parent (mother or father) completed the questionnaire, they provided the socio-demographic information for the other parent. Table 1 presents the parents' socio-demographic characteristics. Of the participating parents, eleven originated from countries outside the Nordic region. All possessed an educational level corresponding to compulsory schooling or higher.

The number of children was 72. The two groups were similar in total sample size (AML 2012: *n* = 34; vs. B-NHL: *n* = 38), but the number of boys was higher in both groups. Table 3 shows the children's characteristics.

### 3.2 Quantitative results

The factor analysis based on the nine items in the questionnaire uncovered underlying relationships between measured variables, ending up in three factors and one indicator. An indicator is an item with a strong independent loading, and one item in the analysis displays this characteristic (Tables 4–7).

#### 3.2.1 Influence of given information

Factor 1 (items 1, 2 and 6) represents the informed consent process, the parents perceived satisfaction with the information, and the understanding of the ICP. Most parents, regardless of their child's diagnosis, stated that they had received enough information. Still, there were signs of uncertainty in both groups as approximately 20% of the parents were “not sure” if they had understood the information (Table 4).

#### 3.2.2 Emotional influences

Factor 2 (items 3, 7 and 8) stands for the perceived degree of voluntariness and the pressure the parents felt during the ICP. This related to parents' perceived knowledge regarding whether their child was involved in the ICP or not. Most of the parents did not feel any pressure regarding enrollment in the RCT. In the AML 2012 group, almost half of the parents meant that their child was involved in the ICP, and the other half said the opposite. In the B-NHL 2013 group one third of the parents responded that the child was involved in the ICP and more than half of them said that the child was not involved in the ICP (Table 5).

#### 3.2.3 Influence on decision-making

Factor 3 (items 4 and 9) shows the parents’ reflections on the ICP; if they felt that they had enough time to consider the treatment options RCT or standard treatment. Although the interval from AML diagnosis to initiation of therapy is typically measured in hours rather than days, most of the parents in the AML 2012 reported that they had had sufficient time to consider their child's enrolment. Whether they would make the same decision again if asked to enroll their child in a RCT at this moment was also reflected. The majority responded that they would make the same decision again. The parents in the B-NHL 2013 were less certain about whether they had had sufficient time to consider the available options, as one-third either responded “No” or “not sure” (Table 6). A similar pattern of uncertainty emerged in the subsequent open-ended question, that is whether they would make the same decision again.

#### 3.2.4 Perceived impact on care in the event of non-participation

The Indicator (item 5) refers to the parents' perceived knowledge of what would happen if their child had not enrolled in the RCT. This reflects the important aspect of understanding what the available treatment options are if the family chooses not to participate in an RCT. Approximately half of parents were confident that the child would receive the same medical care. However, as many as one-third of the parents were uncertain whether non-participation would make a difference to the child's medical care (Table 7).

There were no statistically significant differences between protocols for any of the variables examined, that is, Factor 1 (*p* = .570), Factor 2 (*p* = .308), Factor 3 (*p* = .017), or for the Indicator variable (*p* = .174), as assessed by Fisher's exact test.

### 3.3 Qualitative results

The qualitative analysis resulted in three themes, *Opportunities and risks with new treatment, Contributes to research/ development and help others,* and *Information and strains in the situation* – which together explain the motivations for enrollment.

#### 3.3.1 Motivation for enrollment

In the written statements, that is the responses to the open-ended questions, the parents gave the most important reasons for letting their child enroll in an RCT and what the reasons could have been if they had denied enrollment in the RCT. The themes include both positive and negative consequences which are presented below.

The theme *Opportunities and risks with new treatment,* the parents described how the enrollment in an RCT gave them an opportunity to “*give [name] the best treatment*” or as several statements said, “*give my child a chance of better treatment*”. On the other hand, the risk of being part of an RCT was also highlighted, as some stated “*the experimental arm should give less effect/more side effects*”. Another parent wrote “*The idea of ‘drawing lots’ to determine the care situation, and thereby the child's future*” was a negative consequence of the treatment being an RCT.

The theme *Contributes to research/ development and helps others* reflects the altruistic side of the parents’ thoughts as many of the parents have written similar statements. A reason to enroll their child was “*Wants to be involved in developing the most effective treatment protocol.* “ Another statement was “*So that research can move forward”*. A common statement among the parents was “*helping other children*” or “*To contribute to helping other children in similar situations*.”

The theme *Information and strains in the situation* mirrored some of the information the parents have considered. The question on what the reason to deny enrollment in an RCT could have been, was commented *“don't know. It was clear to me that participating in the study was the obvious choice. I didn't hesitate for a moment.”* Although, parents referred to the possible burden an enrollment and participation in an RCT could be, “*stressful life situation*” and “*increased workload.”* There were also referrals to treating pediatric oncologists' opinion. One statement about why participating was “*The doctor's opinion and the information provided by the doctor.”*

### 3.4 Intertwined quantitative and qualitative results

The quantitative and qualitative findings converged in several meaningful ways. Factor 1, reflecting the centrality of information, together with Factor 2, capturing perceived voluntariness and emotional pressure, corresponded well with the qualitative theme “*Information and strains in the situation.”* Parents emphasized the need for clear and comprehensible information while simultaneously navigating emotional stress and contextual constraints during the consent process. Parents expressed wishes to contribute to research, support clinical development, and help others. The qualitative theme “Contribute to research/development and help others” was mirrored quantitatively in Factor 3, which indicated a motivation to influence and support research despite the challenges posed by the situation. Furthermore, the distinct Indicator highlighted the importance of acknowledging parents' concerns regarding both opportunities and risks associated with novel treatments. This aligns with the qualitative theme “Opportunities and risks with new treatments.” The qualitative findings can be summed up as a motivation for enrollment which captured how parents reasoned about their decision, the emotional and contextual factors shaping their judgments, and their perspectives on their child's involvement in the ICP, which in general illustrate the findings of this study.

## 4 Discussion

This Nordic cross-sectional study used a study-specific questionnaire to examine parents' experiences of the ICP when their child was offered participation in an RCT as a treatment option within either the AML 2012 or the B-NHL 2013 protocol. The findings were comparable across the two RCTs, indicating that despite differences in diagnoses, parents reported similar experiences of the ICP. The factor-analytic results – encompassing information needs, emotional aspects, decision-making dynamics, and considerations related to novel treatments –aligned closely with the qualitative themes regarding motivation for enrollment, irrespective of diagnosis.

The present study revealed that most of the parents included were of Nordic origin, and all parents had completed at least compulsory education, corresponding to nine years of schooling, that is the parents were generally able to read and understand the provided information. The results indicate that the parents felt informed and comfortable with the information process, shown in Factor 1, which captured the impact of information given. However, some parents highlight that the information of what is an RCT and what is an established treatment could be hard to grasp. This highlights a persistent challenge in pediatric trials (12) where emotional stress and limited familiarity with research concepts may hinder understanding. Clinically, these findings underscore the need for clearer and layered explanations of randomization and treatment uncertainty (22). Strengthening communication practices in this way may support more genuinely informed and less burdensome decision-making, even though most parents in our study were satisfied with the ICP and saw no reason to withhold consent for enrollment. These results correspond well with previously reported Nordic research in the field (11, 19). The present study findings were further supported by the written free-text comments, which formed the basis for the qualitative theme *Information and strains in the situation*. Still, 22% of the parents expressed uncertainty about whether they were satisfied with the information provided to their child, suggesting that comprehension may not have been complete. This discrepancy highlights the complexity of ICP in RCTs in emotionally charged contexts, such as pediatric oncology. Studies have reported that parents often struggle to fully comprehend the entire ICP while simultaneously making decisions in the context of their child's life-threatening diagnosis (10, 11). Stress, cognitive load and language barriers can impair individuals when processing information (23, 24). It underscores the need for strategies such as the role of stepwise providing information and obtaining consent and asking the person to repeat the information in his/her own words.

In the current study, parents expressed that the views of the physician played a significant role in shaping their decisions about RCT participation. It is noteworthy that in previous studies conducted in Sweden, a Nordic country, healthcare professionals within pediatric oncology have been examined regarding their management of the ICP (25, 26). These studies revealed that healthcare professionals were concerned that the information may overwhelm the parents and the burden of making decisions in a life-threatening situation may simply be too great (25, 26). The present research may reassure healthcare professionals that despite the inherent challenges of the ICP, most parents are able to understand and handle the demands of the situation. However, healthcare professionals should remain attentive to each parent's individual reactions and needs.

The degree of voluntariness and the child's involvement in the ICP, from the parent's perspective, is presented in Factor 2. These results reflect parents’ perceptions of voluntariness and child involvement in the ICP, indicating that family dynamics –meaning the interactional patterns, decision-making roles, and emotional context within the family –may play a role in shaping how the consent process is experienced. However, the extent to which the parental decision was voluntary is difficult to determine from our results. The feeling of pressure can be both internal and external. Internally, the parental urge to “do something” (14), was evident in theme *Opportunities and risks with new treatment*. External pressure could stem from the influence of the pediatric oncologist, a person whom the parents trust (14, 27, 28). This trust was also reflected in the theme, *Information and strains in the situation*, where the parents referred to the pediatric oncologist's opinion when making the decision.

This study does not involve children's experiences *per se* and there are no written statements in the questionnaire regarding the child's engagement in the ICP. Consequently, we cannot determine how, or to what extent, the child was actually involved. Good Clinical Practice advocates that the child should be included in the ICP and provide assent to the RCT, depending on the child's level of maturity (13). It is not specified what information was communicated to the child, apart from the written age-appropriate information provided in both RCTs. However, the study results show that more than half of the parents were satisfied with the information the child received, Conclusions drawn from the parental ratings in the questionnaire and from the written statements suggest that parents remain the primary decision-makers regarding treatment decisions – circumstances that have been highlighted previously (16). In accordance with the literature, children tend to be more active in shared decision making when the issues address aspects of their daily activities (16, 29).

Parents' responses regarding their perceived influence on the consent process, specifically the amount of time they were given to decide about participation in the study and whether they would make the same decision if asked again, are summarized in Factor 3. The study results showed a non-significant difference between the treatment protocols and the parents' reported responses. When parents were asked about participating in an RCT at the time of their child's acute illness the majority reported, independent of the child's diagnosis, that they had sufficient time and felt confident in their decision. On the other hand, approximately one in five parents responded either with no or was not sure if they felt they had been given sufficient time to consider participation. A similar pattern of uncertainty between diagnoses emerged in the subsequent open-ended question regarding whether they would make the same decision again. However, there was some variation between the protocols; that is, parents of children treated for AML were more likely to respond affirmatively compared with parents of children treated for B-NHL, although the difference was not statistically significant. This finding may reflect differences in clinical routines regarding the amount of time allocated for parents to consider participation in the RCT. It may also be attributable to variations in how the written information was formulated, including the degree to which the language was accessible to individuals without medical knowledge. Previous research in other pediatric specialties involving parents, such as in neonatal intensive care and those with neurodevelopment disorders (24, 30) has shown that parents may view their child's participation in research differently depending on the acuity of the child's condition when they are approached about enrollment. In the present study, both diagnoses (AML and B-NHL) are defined as life-threatening. During acute phases, when parents often experience shock and an intensified perception of illness severity, their reliance on clinicians may become heightened and contribute to more confident decisions about research participation (24).This pattern was confirmed in the present study within the theme *Information and strains*.

The statements on the theme *Opportunities and risks with new treatment* addressed aspects that were not captured by the questionnaire items. These statements revealed parents' reasons for and concerns about enrolling in the study. The primary motivations for enrollment were the desire to access potentially better treatment, and concern was the fear of regretting the decision, particularly if the outcome was poor – are consistent with findings reported by other parents in similar contexts (15). Other have reported that parents remain at peace with treatment decisions made for the child over time, although decisional regret is not absent and affected a notible minority (31). Secondary motivations included altruistic considerations, such as contributing to the development of improved therapies for future children and supporting advancements that may benefit the next generation of patients (15, 27).

The indicator *Perceived impact on care in the event of non-participation* did not follow the same pattern as the other items within each Factor, as these responses appeared to reflect parents' speculations about what might happen if they declined to enroll their child. Existing research suggests that patients often hesitate to go against a physician's wishes, as they fear it might result in poorer care or treatment (27).Therefore, physicians and other healthcare professionals involved in the ICPs must be aware of how, when, and by whom information is communicated, as these factors clearly influence the decision-making and motivation to participate in RCTs. The situation surrounding a cancer diagnosis – characterized by emotional pressure and a short timeframe, in which decisions must be made – inevitably affects how parents act. That said, this study has not investigated parents who declined participation or their reasons for doing so. Prior research has reported that parents who have declined to enroll their child in an RCT in pediatric oncology felt that the decision was experienced as burdensome (19). In neonatal care, parents have expressed a preference for the clinical treatment team – rather than researchers alone – to be involved in the process (24). In Nordic pediatric oncology settings, healthcare professionals often hold dual roles, meaning they are responsible for both clinical care and the conduct of research studies (25). This approach carries the risk of what is referred to as therapeutic misconception (32), that is, the study participant believes that the primary purpose of an RCT is to provide individualized therapeutic benefit, rather than to compare treatments under controlled conditions to generate generalizable scientific solid knowledge. When dual clinical and research roles are employed, they must be managed with great care to ensure clarity of role boundaries and prevent ethical concerns (17).

## 5 Strength and limitations

A strength of our study is that the participants were drawn from four of the five Nordic countries, forming a larger cohort representing countries and multiple languages. Furthermore, a strength is the approach with multimethod analyses of both quantitative data and qualitative data. Using a multimethod approach, including free-text responses, the entire dataset was analyzed, contributing to a deeper understanding of the phenomenon studied. This study design facilitated a more in-depth understanding of parents' experiences and thoughts of the ICP and their motivation for letting their child enroll in an RCT. Another strength of the study is the comparison of the parents' experiences across two different diagnoses in pediatric oncology, both involving phase III RCTs with similar treatment durations and comparable treatment designs.

The limitations include that only parents whose children were enrolled in an RCT were invited and we did not study parents who declined participation. When questions are asked retrospectively, there is an inherent risk of recall bias. An important limitation is the absence of children's own views, which need to be explored in future research.

## 6 Conclusions

In pediatric oncology, phase III RCTs frequently constitute a first-line treatment option. This study contributes to a broader understanding of parents' experiences of the ICP within the context of standard treatment practice. Despite making decisions under considerable emotional pressure, most parents reported satisfaction with the information provided and did not express regret about their enrollment decision. The findings underscore the relevance of communication practices that support parents' comprehension and decision-making during the consent process. Future research should address children's involvement in the ICP from diagnosis through treatment and follow-up. These efforts should engage children in ways that reflect their developmental stage and linguistic and cultural background, while still allowing them to defer the decision to their parents when appropriate.

## Data Availability

The original contributions presented in the study are included in the article/Supplementary Material, further inquiries can be directed to the corresponding author.
